# Student Perceptions of Preparation and Competency Development During Extramural Clinical Rotations in Germany: An Online Survey †

**DOI:** 10.3390/vetsci13070642

**Published:** 2026-06-30

**Authors:** Sandra V. Kielmann, Roswitha Merle, Jörg R. Aschenbach, Annika Fels, Katharina Charlotte Jensen

**Affiliations:** 1 Institute of Veterinary Epidemiology and Biostatistics, School of Veterinary Medicine, Freie Universität Berlin, 14163 Berlin, Germany; 2 TVD Finanz GmbH & Co. KG, 28816 Stuhr, Germany; 3 Institute of Veterinary Physiology, School of Veterinary Medicine, Freie Universität Berlin, 14163 Berlin, Germany

**Keywords:** veterinary education, practical year, extramural clinical rotations, evaluation, clinical placements

## Abstract

Extramural clinical rotations (ECR) are an integral part of veterinary education in Germany and give students the opportunity to practice and deepen their knowledge and skills. In this study, students from all five veterinary schools in Germany who completed their practical year between the years of 2022 and 2025 were surveyed online regarding their perceived preparedness, their evaluation of teaching during ECR, and what aspects were relevant to them when choosing ECR. Data records of 386 students were analyzed. Being assigned a direct supervisor and feedback meetings were among the five most important aspects for students when choosing their ECR, underlining the importance of a clear structure and constructive feedback for successful learning. More than half of the students reported feeling insufficiently prepared, both theoretically and practically, for surgery. Although the students already passed their propaedeutics exams years before, only 46% of respondents stated having felt (very) well prepared in terms of propaedeutics knowledge going into their ECR, 59% felt (very) well prepared in terms of performing general examinations (routine clinical examinations) and 20% in terms of specific examinations (e.g., neurological or rectal examinations). Meanwhile, 75% of students rated the quality of practical teaching during their ECR positively. However, in some areas—such as specific examinations and diagnostic imaging—there was still room for improvement. Overall, the results suggest that ECR are generally perceived positively. Concerning the feeling of mostly insufficient preparation, further studies are needed to elaborate whether teaching should focus more on practical skills.

## Simple Summary

Theoretical knowledge and practical skills are a vital part of working as a veterinarian. Students in Germany learn these skills during their studies at universities and during their practical year, which takes place in their last year of education. In this time, students complete extramural clinical rotations in veterinary practices, where they work with real patients and have the opportunity to apply and further develop their practical skills. These placements are intended to help students prepare for their future work as veterinarians. To benefit as much as possible from this period, it is important that students are well prepared before starting their practical year. However, the results of this study show that a significant percentage of students felt insufficiently prepared regarding many aspects of their theoretical knowledge and practical skills. In contrast, the teaching during the extramural clinical rotations was mostly perceived positively. Although there is still room for improvement, this study indicates that these placements provide valuable opportunities for hands-on learning and practical training. The study also suggests in which areas additional training may be beneficial, helping students feel better prepared for their extramural clinical rotations. Further studies are needed that include not only the students’ point of view.

## 1. Introduction

Extramural clinical rotations (ECR) are an important tool in the education of veterinary students [1,2], as they provide students with the opportunity to apply and deepen their knowledge and clinical skills, and develop interpersonal skills [3]. During their ECR in private practices or clinics, students encounter a higher caseload and gain practical experience in areas such as practice management, teamwork and leadership, economics, time management, and client communication [4]. As most veterinarians work in clinical practice [5], it is important for veterinary students to become familiar with the typical daily workflow and the types of cases commonly encountered in this setting. While a lot of private practices handle routine first-opinion clinical cases, they might refer more complex cases to referral hospitals, including the university-based teaching hospitals. During their intramural clinical rotations, students might see more of those difficult cases, and therefore miss out on the normal day-to-day cases that they will encounter in private first-opinion practices [6]. Intramural training is governed by a mandatory catalog of clinical rotations. By contrast, ECR were long reliant on recommendations by the veterinary education establishments due to a lack of specific requirements, which made regulating these placements difficult [7]. A framework for the extramural clinical rotations was only created in summer 2023 with the release of the new European System of Evaluation of Veterinary Training Standard Operating Procedure (ESEVT SOP) [8].

Good preparation is essential for a successful ECR, as it affects the students’ ability to engage during their clinical rotations and take advantage of learning opportunities, which play a key role in developing the competencies necessary for graduation [9] and for a successful transition into professional life. From a workplace learning perspective, learning in clinical settings is shaped by the interaction between students’ prior preparation and the learning opportunities provided in authentic work environments, where active participation is central for competence development [10]. In this context, clinical knowledge such as propaedeutics—one of the first clinical courses, where students learn basic animal handling and clinical examination skills and knowledge—provides fundamental skills for veterinary assessment and therapy and is therefore crucial for effective practice. Similarly, knowledge of common diseases—how to diagnose and treat them—is part of every case presented to veterinarians. Failure to identify certain diseases can be dangerous, not only for the animal but also for everyone involved in the case, as it poses the threat of disease transmission or spread. From a competency development perspective, this process can also be related to Miller’s framework of clinical competence, in which knowledge acquired during pre-clinical training (“knows” and “knows how”) is applied and demonstrated during workplace-based learning such as ECRs (“shows how” and “does”) [11]. Although preparation is a subjective term and might be perceived differently among individuals [12], there is a clear tendency for students to report feelings of insufficient preparedness for their ECR [13]. Nevertheless, the ECR is widely regarded as a positive experience [7] and students perceive an improvement in their Day-One Competencies during this time [3].

In Germany, the veterinary medicine study program is organized into three stages. The first, called the pre-clinical stage, covers the first four semesters. The second stage, also referred to as the clinical stage, starts in the fifth semester, and includes courses such as microbiology, pharmacology, and pathology, as well as more directly clinically linked courses such as surgery and internal and reproductive medicine. During this time, students develop clinical key competencies for the following stage. The third stage consists of the Practical Year (PY), which takes place during the 9th and 10th semesters, and leads up to the 11th semester, during which the final exams take place.

The PY consists of multiple work placements. These include a total of seven weeks in veterinary public health institutions (VPH), an intramural clinical rotation of at least ten weeks conducted at a university clinic or institute, and an ECR completed outside the university. The extramural clinical part consists of a four-week clinical rotation at a veterinary practice or clinic, which students are free to complete starting after the fifth semester during their lecture-free periods or during their PY, and another sixteen weeks divided into two halves: eight weeks have to be completed at a veterinary practice or clinic and an eight-week elective placement that may be carried out in any veterinary-related setting including clinical practice [14].

The practices in which ECR take place must meet certain requirements: the presence of a veterinarian with at least two years of independent professional experience and the absence of professional disciplinary sanctions within the last two years, as well as an operational in-house pharmacy [14].

In 2023, the European Association of Establishments for Veterinary Education (EAEVE) released its new ESEVT SOP, in which the quality assurance (QA) of ECR gained due attention [8]. The QA measures of ECR include a clinical rotation agreement between the university and the veterinary practice or clinic in which students complete their extramural clinical rotations, a logbook in which learning objectives are defined—based on institutional or curricular templates—and their achievement is documented, a monitoring system to ensure the quality of the clinical rotations by evaluating them and giving feedback and, lastly, the supervising veterinarians must complete a one-time didactic training of at least 4 h [15]. To support these QA measures, the German Veterinary Medicine Faculty Council (VMFT) has launched an online platform—the VMFT-Service-Center—where these QA measures are integrated for low-threshold implementation by the training veterinarians (https://www.vetmed.fu-berlin.de/en/studium/vmft-service-center/index.html (accedded on 29 April 2026)). However, as the new regulations and the VMFT-Service-Center have not been in place for long, little is known about whether students actively consider these criteria and to what extent the VMFT-Service-Center is used when selecting their ECR.

While students are mostly assigned to their intramural clinical rotations, they must organize the rest of the PY themselves. This includes placements in VPH as well as the ECR. This places significant organizational demands on the students, as they must secure their placements, sometimes considering desired time frames, and eventually arrange financing, accommodation, and transport. The transition from university to the outside world and to working practices and clinics where students complete their clinical rotations can be a stressful time period for students. During this time, they might feel anxious about having to demonstrate their knowledge [9], while other potential stress factors include finances and accommodation [16]. Previous studies have explored the factors influencing students’ decisions on where to complete their ECR, with placements that could be considered as future employment, clinical specializations that students might be interested in, the prospect of future clinical specializations in a certain field, financial considerations, and accommodation arrangements being named as criteria [13,17]. Another factor was “finding a practice that would provide opportunities to develop clinical practical skills” [17], whereas the proximity to home varies, being considered important in some studies [17] and of less significance in others [18]. However, despite these insights, little emphasis has been placed on how students themselves rank the importance of certain criteria—specifically, which are more important and which are rather irrelevant, and whether said criteria are met.

Against this background, we conducted a survey among German veterinary students in order to answer the following questions:

1. Which selection criteria do students apply when choosing their extramural clinical rotations and to what extent are these criteria met?
2. How well-prepared do students feel for their practical year in terms of various theoretical knowledge and practical skills?
3. To what extent are the mentioned knowledge and skills taught during their extramural clinical rotations?
4. How do students perceive the organizational and financial aspects of the practical year?
5. To what extent are the overall expectations of their extramural clinical rotations fulfilled?

## 2. Materials and Methods

### 2.1. The Survey

The study was approved by the Central Ethics Committee of the Freie Universität Berlin (No. VI B 2; approval no. ZEA25010) and conducted in compliance with German and European data protection regulations. Participants had to provide informed consent on the survey landing page.

Initially, the literature was reviewed to identify relevant domains and constructs related to students’ preparedness and satisfaction. In addition, evaluation forms from all five veterinary schools were reviewed to include existing assessment approaches and commonly used items. Based on this, hypotheses and questionnaire items were defined. A questionnaire was created and implemented into LimeSurvey© Cloud (LimeSurvey GmbH, Hamburg, Germany). The questionnaire consisted of a total of 17 questions, although not every participant had to answer them all, as answers to the first two questions determined their eligibility to continue. The survey included 14 mandatory questions: four single-choice (SC), one multiple-choice (incl. one free text option, MC), one percentage allocation question (sum 100), and eight Likert-scale questions (5-point scale, two with the option “no answer”). Furthermore, there were three open questions that were voluntary: two to specify or add information to their answers to the prior questions, and one at the end to give the participants the possibility to voice their opinions on their experience during the practical year.

The questionnaire was divided into three sections. In the first section, questions were asked about the university they attended and the years in which they completed their practical year. In the second section, participants provided information on the animals they treated during their ECR, which selection criteria were relevant when looking for an ECR, which of those were fulfilled, and how they found their placements. The third section focused on a self-assessment of students’ theoretical knowledge and practical skills at the start of the practical year, and how well said knowledge and skills were taught during their ECR. It also addressed financial and organizational challenges, as well as overall satisfaction with their practical year and ECR. The questionnaire is provided as part of the Supplementary Materials.

To improve the quality of the survey and assess comprehensibility, a pre-test was carried out with people from different groups (professors, veterinary students, veterinarians, and non-veterinarians; *n* = 14). Minor changes were made, and the survey was activated on 27 June 2025. The target group consisted of students from all five German veterinary medicine institutions who had started their PY in the winter semester of 2022/2023, 2023/2024, or 2024/2025. As previous cohorts potentially experienced greater limitations during their practical year due to COVID-19 measures, they were not included in the target group. The (former) students were invited to partake in the survey via a link sent out by email by the TVD Finanz GmbH & Co. KG and the general student committee (Allgemeiner Studierendenausschuss) of the University of Veterinary Medicine Hannover. It was further distributed via the social media channels (Instagram, Facebook) of the German Veterinary Students Association (bvvd e.V.), the International Veterinary Students Association Germany (IVSA Germany), the TVD Finanz GmbH & Co. KG, as well as through several groups on Facebook and WhatsApp. Here, closed groups including the mentioned classes were used. Participation was voluntary, and participants were informed of the study’s aims and the opportunity to withdraw from the survey at any time without consequences. They were also given the option to enter a prize draw at the end of the survey to win a tumbler or one of two EUR 25 vouchers. The prizes were sponsored by the TVD Finanz GmbH & Co. KG. The survey was closed on 25 September 2025.

### 2.2. Statistical Analyses

No IP tracking or other technical measures were used to identify or prevent duplicate responses. Partially completed questionnaires were included in the analysis, and available responses were evaluated for the respective questions. Missing data were handled by analyzing each item based on the number of respondents who provided an answer.

The statistical analysis was carried out using IBM SPSS Statistics (Version 31). Only responses from (former) students who had studied in Germany and had completed their practical year mostly in Germany, starting in the winter semesters of either 2022/2023, 2023/2024, or 2024/2025, were included. To determine the total number of students in each semester at the different universities, each university was contacted and provided the respective numbers. Participants who dropped out before question 6 were excluded from the evaluation. Some free-text answers concerning the methods of finding their ECR placement and the relevance and fulfillment of criteria were sorted into corresponding categories. Relevant categories were combined where required; for example, ruminants, pigs, and poultry were combined into the category livestock. First, descriptive analyses were carried out, including frequencies and percentages. The question regarding the percentage of species examined or treated was then analyzed in two ways: first, whether a species was treated at all (multiple responses possible) and, second, whether there was a focus on a single species or a defined group of species (e.g., livestock), defined as ≥75%. Differences between groups differing in financial aspects were assessed using the chi-square test (CS). Differences between cohorts (PY 2022/23, PY 2023/24, and PY 2024/25) were assessed using the Kruskal–Wallis (KW) test to explore potential recall bias. To test if the perception differed among the single skills, Friedman’s rank tests with post hoc tests and Bonferroni-adjustment were calculated for (a) preparedness in terms of knowledge, (b) preparedness in terms of skills, (c) teaching in terms of knowledge, and (d) teaching in terms of skills. The results are mainly presented graphically using histograms and stacked centered bar charts, which were created using Microsoft Excel^®^ (Version 16.106.3), SPSS, and R 4.6.1 [19] with RStudio [20] and the package ggstats [21].

## 3. Results

### 3.1. Demographics

A total of 496 people opened the link to the survey, and 110 data records corresponding to people who were not part of the target group (*n* = 16) or who failed to complete most of the survey (*n* = 93), and one person who spent most of their practical year abroad were excluded. Therefore, 386 data records were finally analyzed, including 11 that were partially incomplete, with the provided answers still contributing to the evaluation. The response rate was 14%.

**Figure 1:**
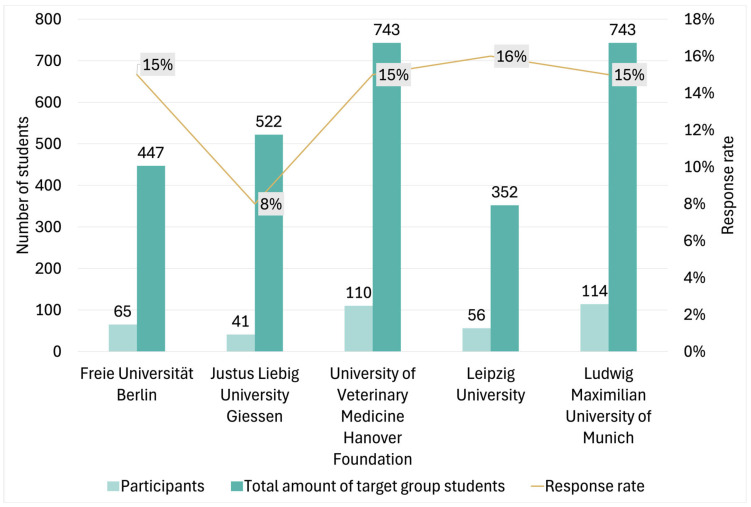
Number of participants out of the total number of target group students at each German university (*n* = 386) and the resulting response rate.

Of the participants, 30% (114 of 386)—and, thus, the largest group—attended Ludwig Maximilian University of Munich, closely followed by students from the University of Veterinary Medicine Hannover Foundation: 28% (110 of 386). Furthermore, 17% (65 of 386) were from Freie Universität Berlin, 15% (56 of 386) from Leipzig University, and 11% (41 of 386) from Justus-Liebig-Universität Giessen (Figure 1). Concerning the surveyed cohorts, almost half (49%, 190 of 386) undertook their practical year starting in the winter semester 2024/2025, 30% (117 of 386) started in the winter semester of 2023/2024, and 20% (79 of 386) in the winter semester of 2022/2023.

### 3.2. Placement Search and Selection Criteria

**Figure 2:**
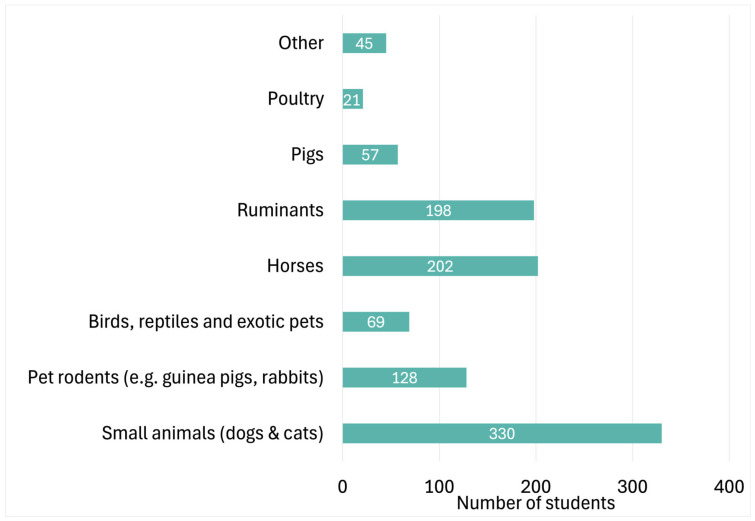
Animal species students treated during their ECR (multiple answers possible; *n* = 386).

Figure 2 shows which animals were treated by students during their extramural clinical rotations. Of the students, 37% (143 of 386) treated primarily companion animals (incl. dogs, cats, rabbits, birds, reptiles, etc.), with at least 75% of their cases involving companion animals. Additionally, 12% (46 of 386) treated mainly horses and 8% (32 of 386) treated mainly livestock (ruminants, pigs and poultry).

The participants’ different methods for finding their placements are shown in Table 1. Notably, none of the respondents reported using the VMFT-Service-Center to identify ECR placements, despite it being one of the available options listed in the survey. Participants who chose “other” had the option to specify this, with 16% (61 of 386) reporting prior familiarity with the practice (e.g., through previous employment, as their own pet’s veterinarian or through friends and family), and 4% (17 of 386) had used web search engines such as Google to look for practices; for example, in their local area.

**Figure 3:**
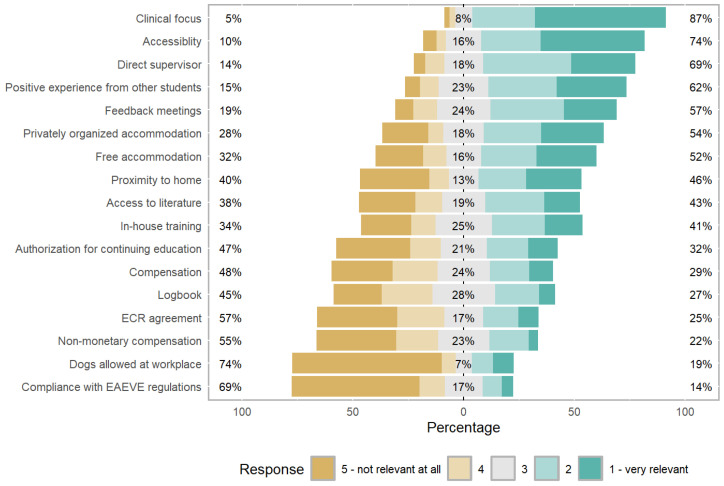
Relevance of certain criteria during ECR (no response possible): Clinical focus (*n* = 382), Accessibility (*n* = 385), Direct supervisor (*n* = 386), Positive experience from other students (*n* = 382), Feedback meetings (*n* = 382), Privately organized accommodation (*n* = 358), Free accommodation (*n* = 371), Proximity to home (*n* = 385), Access to literature (*n* = 380), In-house training (*n* = 381), Authorization for continuing education (*n* = 378), Compensation (*n* = 374), Logbook (*n* = 377), ECR agreement (*n* = 378), Non-monetary compensation (*n* = 366), Dogs allowed at workplace (*n* = 321), Compliance with EAEVE regulations (*n* = 329).

Figure 3 shows the selection criteria that were more or less relevant when looking for their ECR placements. Means of accommodation seemed to be a rather polarizing factor, with almost half the participants (46%, 179 of 385) putting high importance on the proximity to home, whereas 40% (155 of 385) found it less important. In contrast, both free accommodation provided by the practice and privately organized accommodation through friends or family were rated as slightly more important. Accessibility (e.g., by public transport or private car) was generally considered important. Overall, the most important factor was the practice’s clinical focus aligning with the students’ own professional interests. In an open question, students had the opportunity to list other criteria they valued: 6% (24 of 386) mentioned social factors such as team atmosphere and likeability, and 5% (19 of 386) placed high relevance on practical training with the opportunity to gain hands-on experience.

**Figure 4:**
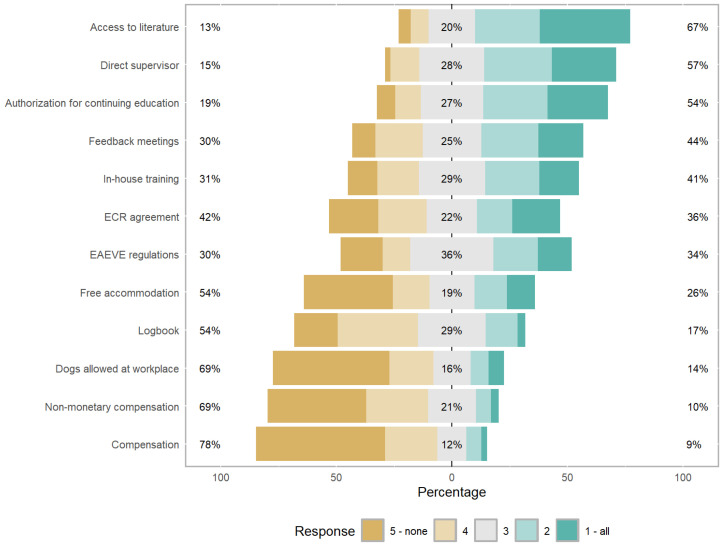
Fulfillment of certain criteria during ECR (no response possible): Access to literature (*n* = 370), Direct supervisor (*n* = 375), Authorization for continuing education (*n* = 352), Feedback meetings (*n* = 375), In-house training (*n* = 367), ECR agreement (*n* = 376), Compliance with EAEVE regulations (*n* = 196), Free accommodation (*n* = 370), Logbook (*n* = 376), Dogs allowed at workplace (*n* = 222), Non-monetary compensation (*n* = 375), Compensation (*n* = 376).

Figure 4 shows whether these criteria were fulfilled. In the question on the fulfillment of the criteria, factors were excluded if the workplace had no direct influence (recommendations of other students, proximity to home, accessibility, private accommodation, and areas of professional interest).

When asked about the financial burden of the practical year, only 18% (68 of 375) reported little to no burden, and approximately a quarter (25%, 92 of 375) reported a moderate burden, while 31% (116 of 375) experienced a considerable financial burden and 26% (99 of 375) reported a substantial financial burden. Those experiencing a considerable or substantial financial burden also reported being rather dependent on financial compensation (54 of 215; CS: *p* < 0.001). Similarly, those relying on financial compensation placed a higher priority on receiving one when selecting their ECR (47 of 63; CS: *p* < 0.001). Some students (*n* = 4) indicated that the reason for not prioritizing receiving compensation during their ECR was that very few placements actually provide a wage for ECR students. Perceived financial burden differed significantly between cohorts (KW: *p* = 0.046; Supplementary Table S1), with students from the more recent cohorts reporting slightly higher levels of financial burden than students from the earliest cohort.

**Figure 5:**
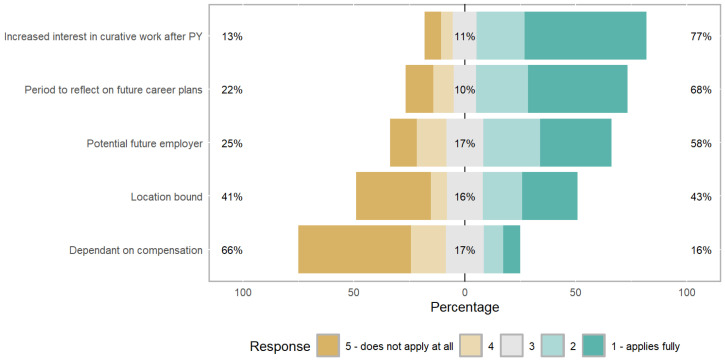
Students’ opinions towards their ECR and agreement with factors influencing their ECR (*n* = 385).

Figure 5 shows students’ agreement with their perceptions of their PY and different factors that further influenced their ECR and the selection thereof.

### 3.3. Preparedness for and Provision of Theoretical Knowledge and Practical Skills During the Practical Year

**Figure 6:**
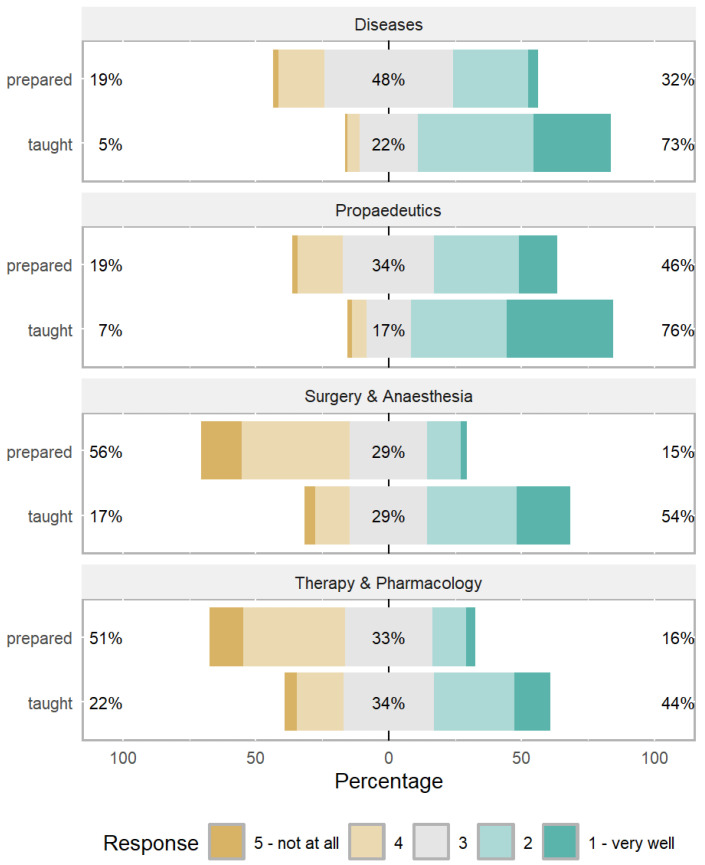
Students’ assessment of theoretical preparedness before their PY and theoretical teaching during their ECR (*n* = 375).

**Figure 7:**
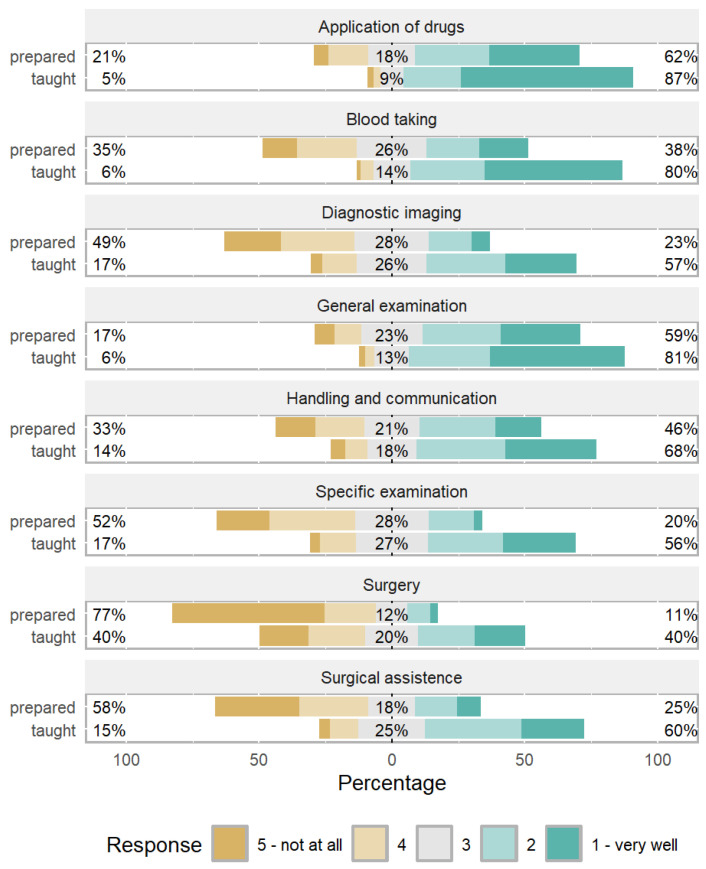
Students’ assessment of practical preparedness before their PY and practical training during their ECR (*n* = 375).

In Figure 6 and Figure 7, the perceived preparedness and the students’ assessment of the teaching of knowledge and skills are displayed in order to examine the distribution of responses across both dimensions for each competency. In terms of theoretical knowledge, students felt best prepared in propaedeutics (basic animal handling and clinical examination skills and knowledge) and common diseases (symptoms, etiology, and diagnostics; *p* < 0.001; Figure 6 and Supplementary Figure S1), whereas the perceived preparedness was worse for therapy and pharmacology (active substances, dosage, and dosage calculation), as well as surgery and anesthesiology (Figure 6). While students rated the teaching of these topics during their ECR noticeably higher (Figure 6 and Supplementary Figure S2), the same overall pattern persisted: propaedeutics received the best ratings, and therapy and pharmacology the worst (*p* < 0.001). For the perceived preparedness, application of drugs (medication administration; e.g., intramuscular or subcutaneous injections) and general clinical examination were rated the best, followed by blood-taking and handling and communication. Regarding diagnostic imaging, specific examinations (specialized clinical examinations; e.g., neurological or rectal examinations), and surgical assistance, preparation was assessed better than that for surgery (*p* < 0.001; Figure 7 and Supplementary Figure S3). A similar ranking of skills was observed in the students’ assessments of the teaching of the mentioned skills (*p* < 0.001; Figure 7 and Supplementary Figure S4).

To explore potential recall bias, responses were compared across the three cohorts. Significant differences were observed for several preparedness and teaching items, with students from the most recent cohort generally reporting slightly more favorable ratings of both theoretical and practical preparedness and teaching (Supplementary Table S2).

### 3.4. Overall Satisfaction

**Figure 8:**
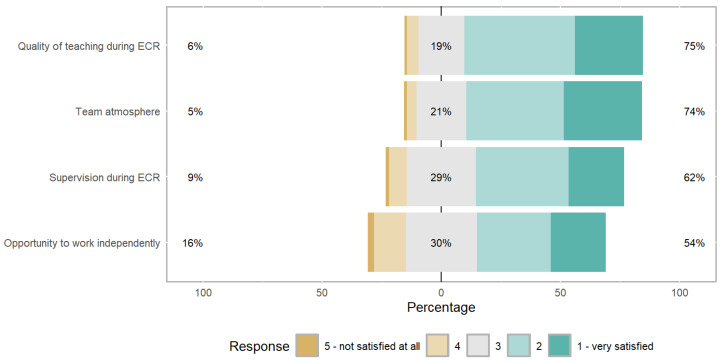
Students’ satisfaction with supervision and working environment during their ECR (*n* = 375).

Of the students, 64% (240 of 375) were able to complete their ECR at both their preferred practices and within their preferred time periods, while 36% (135 of 375) completed it only partially according to their preferences (i.e., either at the preferred practice or within the preferred time period, but not both). The students’ overall satisfaction with aspects regarding supervision and working environment was mostly high (Figure 8). Comparisons between cohorts revealed no significant differences for team atmosphere, supervision during placements, or opportunities to perform tasks independently, indicating largely consistent evaluations across cohorts (Supplementary Table S3).

## 4. Discussion

Overall, students reported varying levels of perceived preparedness across the assessed areas. Regarding theoretical knowledge, preparedness was generally rated lower, whereas theoretical teaching was evaluated more positively across most categories. Concerning practical skills, preparedness varied considerably between categories, while the provision of practical teaching during the ECR was mostly rated positively. There were varying opinions regarding the selection criteria for ECR, and while some criteria were fulfilled more frequently, others were barely met. Generally, the students’ satisfaction with supervision during their ECR was very high.

### 4.1. Limitations

The questionnaire was developed based on a literature review and evaluation forms from all five veterinary schools. However, no formal validation was conducted. Consequently, validity, reliability, and internal consistency could not be assessed. Most constructs were assessed using individual questionnaire items rather than validated multi-item scales, limiting the ability to perform psychometric analyses such as internal consistency testing.

Surveys with convenience sampling generally face limitations in representativity. Nonresponse bias is likely, as students who experienced strong emotions around their ECR might be more inclined to participate in the survey than others. The distribution of the survey via social media may have resulted in an overrepresentation of students who were more engaged with professional organizations or veterinary-related networks. However, as no data on such engagement were collected, the magnitude of this potential bias remains unknown. It remains unknown whether such a bias occurred and, if so, in which direction it may have influenced the results. Since a lot of questions included Likert scales, a skewed distribution known as positivity bias is possible [22].

The survey was made available online starting in June 2025. By that time, the cohorts who had completed their PY in 2022/2023 and 2023/2024 had already graduated, making them substantially harder to reach. This is also reflected by the data: the majority of responses came from the cohort that was still enrolled at university when the survey was launched. In contrast, the 2022/2023 cohort was the most difficult to contact and is therefore the least represented in this study. The most recent cohort was still completing its PY when the survey went online, so their memories were likely more accurate, whereas the two earlier cohorts had already finished their PY and relied on past memories that may have been affected by memory bias. To explore the potential impact of recall bias, responses were compared across cohorts. Several significant differences were observed, particularly for preparedness and teaching ratings. While these findings may indicate an influence of recall bias, it cannot be determined whether they resulted from memory effects, genuine differences between cohorts, or a combination of both.

Of note, this study collected only students’ perceptions of their PY, especially their preparedness before and training during ECR. Therefore, conclusions regarding the actual preparedness and quality of teaching cannot be drawn from it. It is also unknown to what extent participants attended practical courses or used additional training opportunities (e.g., clinical skills labs) or whether prior practical experience (e.g., through previous vocational training) influenced their responses. Further studies are needed to determine whether and to what extent these factors influence students’ perceived preparedness and experiences during ECR.

### 4.2. Selection Criteria

Most students found their extramural clinical rotations through various online channels, particularly by consulting the websites of practices and clinics. This highlights the importance of a strong online presence when advertising student placements. The second most commonly used approach was recommendations from other students, emphasizing the relevance of positive experiences reported by previous students. Only about 11% looked for such recommendations on the bvvd-Website, even though those are student recommendations. The fact that nobody used the VMFT-Service-Center for identification of ECR sites is understandable as this service only went online in the summer of 2024 [23] and opened its voluntary listing of ECR placements in March 2025. By that time, even the last cohort should have had their PY completely planned.

The financing of the PY is an obstacle students must deal with when planning their ECR, and could even limit students’ placement options [17]. Veterinary students have a very high workload [24,25]; a lot of them are regularly employed [25]. Employment is hard to maintain during PY, when they also complete a full workweek for their ECR. For many students, it can be assumed that costs for traveling, accommodation, and the missing opportunity to work besides studying have to be incorporated into their decision on where to undertake these placements [26]. The results indicate that the first cohort assessed the financial burden lower than the following, which might be due to the fact that prices (e.g., for rent) rose substantially within the last three years in Germany or due to positivity bias. The results show that the financial burden was directly associated with their decision-making when choosing ECR, as people who experience a higher financial burden also reported being rather dependent on financial compensation and, thus, limited in choice. While free accommodation was considered important by many students, it was rarely provided. Providing free accommodation could potentially relieve some of the financial and organizational burden from students as planning and financing of accommodation over an extended ECR period can be time-consuming and rather costly.

Riemann et al. [27] showed that more than half the students change their minds or are undecided about their future plans during their studies. This is emphasized by the finding that, for many students, the PY was still a time to figure out what they wanted to do upon entering the workforce (Figure 5). Consequently, some used the opportunity to inquire about ECR at practices or clinics that they considered for future employment, something also shown in previous studies [17]. This provides a chance to work there for a couple of weeks and assess whether the workplace suits them. Börchers et al. [7] stated that work placements are often decisive for future career plans, which is supported by the finding that 77% of students agreed that their ECR increased their interest in curative work after their PY, something that may influence their choice of a curative workplace after graduation.

Some clinics and practices in Germany are eligible to continue postgraduate education for veterinarians, leading to a specialist qualification. The growing importance of specialization [28] is also reflected in the finding that more than half of the students found it at least somewhat important that their ECR workplace was authorized for such specialist training. Availability of in-house training and access to literature might also indicate commitment to continuing education or at least staying up to date on new developments in veterinary medicine. As these measures are relatively easy to implement, it is not surprising that they were frequently fulfilled.

Feedback is a vital part of learning [13], providing students not only with recognition for good work but also guidance on how to improve. Receiving feedback and completing logbooks were found to be very useful to students in previous studies [16]. In this survey, most students rated feedback meetings very high on their priority list, whereas logbooks were less important. About 70% of students received feedback at least at every other ECR placement, whereas more than 50% reported that logbooks were used for fewer than half their ECR. Though some universities had their own logbooks and evaluation forms for students to fill out after completing their ECR, these were not compulsory for the veterinarians in practices and clinics until the introduction of the new ESEVT SOP. While the EAEVE does not mandate a standardized logbook, the logbooks are intended to ensure that Day-One Competencies are achieved [8]. Interestingly, an ECR agreement, though being slightly less important to the students, was more often completed than the logbook. With both being compulsory under the new ESEVT SOP [8], more practices and clinics will have to provide them in the future. While about a third of the students placed at least some relevance on whether their placements were fully compliant with EAEVE regulations, it can be assumed that many did not make it their priority, as they were still in a transition phase and the requirements were not yet compulsory.

Having a direct supervisor supports a structured placement and offers students a clear point of contact, something many students considered important and that was frequently provided during ECR. Providing students with a designated supervisor should therefore be considered an important element of all placements.

### 4.3. Preparedness for and Provision of Theoretical Knowledge and Practical Skills During the PY

Clinical training in a practice or clinic allows students to integrate theory with practice and understand diagnostic and therapeutic processes [29] while also deepening their knowledge and further developing practical skills. Therefore, it is important that students are well prepared for their PY, as a lack of preparation can result in missed learning opportunities [29] and negatively impact their learning outcome [12]. Given this back-ground, it was observed that many students reported perceiving themselves as insufficiently prepared for their PY. This result is consistent with the findings of the study by Drzemalla et al. from 2025 [13], in which 85% of students felt dissatisfied with their practical preparation and 66% with their diagnostic skills before the PY. In particular, regarding special examinations and diagnostic imaging, both studies indicate that students do not feel sufficiently prepared for their PY [13]. Veterinary education in Germany aims to qualify students to become fully trained veterinarians with the ability to practice the veterinary profession across its full scope [14]. While universities should teach students theory and clinical diagnostic thinking, which can then be applied and further developed during their ECR [18], the PY was introduced to improve hands-on clinical training of students through direct patient contact [30]. To do so, students must possess the necessary theoretical competencies, enabling them to apply this knowledge in practice. As shown in Figure 6, students felt they lacked theoretical understanding. Even in propaedeutics, which was rated best among the theoretical topics, fewer than half of the respondents reported feeling well prepared, even though all students were required to have completed the exam in this subject by that time.

Concerning practical skills, this study also indicates that some students perceived their level of preparedness as insufficient. It is plausible that skills that are less invasive, such as general examination, drug administration, and handling of owners and patients, are rated higher than skills that pose a higher risk of failure, such as surgery and surgical assistance. Interestingly, though, special examinations and diagnostic imaging were rated surprisingly low. Since special examinations and diagnostic imaging were not further classified into different options in the questionnaire, it could be argued that students might have rated them so low because they considered all options rather than the main ones most commonly used. For example, a student who has focused on small animal medicine so far might feel unprepared to do an orthopedic evaluation of a horse. Same with students considering advanced techniques, such as magnetic resonance imaging scans, when assessing their preparedness for diagnostic imaging. This is consistent with the findings of a recent study in which students focusing on small-animal medicine rated their own competence in neurological and ophthalmological examinations as rather low [31]. It also confirms another study, in which students were asked to rate their preparedness for different special examinations and diagnostic imaging techniques, and over half the students did not feel confident to perform those tasks [13]. These findings are based on students’ self-assessments and reflect perceived levels of theoretical and practical preparedness. No conclusions regarding actual competence or educational quality can be drawn from this study; further research is needed to address these aspects.

In this context, it is important to mention the possible impact of COVID-19 on university teaching. The propaedeutics course, in which students usually learn the basics of clinical examinations and animal handling and get to practice them on real animals, is allocated to their 4th or 5th semester, depending on the university [32]. This means the first cohort should have started this course in spring 2020, when the first lockdown occurred. Between then and 2022, multiple lockdown periods occurred in Germany, which may have affected the practical teaching at universities and lead to limited in-person courses. Instead, lectures were mostly held online, which posed a challenge to both instructors and students as this format was new and required them to adapt to a different mode of teaching and learning. As a study from 2025 has shown, students perceived this as having a very negative impact on their practical training [27]. However, the present study does not allow conclusions regarding this relationship, and further studies are needed to investigate potential long-term effects.

Previous studies indicate that students’ perceptions of competencies may change as a result of educational experience [33,34]. For example, repeated practice and practical application can lead students to reassess the relevance of certain competencies [33]. Furthermore, the standards against which students assess their own abilities may change as their knowledge increases [34]. This suggests that retrospective evaluations, such as this one, may be influenced by experiences gained in the meantime. Therefore, it is unclear whether students would have rated their preparation more positively had they been asked before their PY. Further studies in this direction are needed to determine how students evaluate their preparedness at different stages of their studies. However, while such bias might have negatively influenced the students’ responses in this study, previous studies from 2025 and 2026 showed that supervisors also perceive students as insufficiently prepared, especially considering practical skills [13,35].

In recent years, the universities in Germany have established clinical skills labs, so students can get some hands-on training in a realistic setting [36]. There, students can repetitively train certain practical skills on simulators which, for ethical and logistical reasons, is not possible on live animals [36,37]. While such training centers provide students with the opportunity to practice their skills, it has been pointed out that some clinical processes cannot be taught on simulators [37]. Therefore, at a certain stage, students must also gain experience with live animals in realistic clinical settings [31], as this reflects their future professional practice. However, this survey did not assess whether and to what extent students used clinical skills labs during their studies. Therefore, it remains unclear whether the use of clinical skills labs was associated with students’ perceived clinical preparedness and no conclusions regarding its impact can be drawn from this study. Further studies are needed to investigate the role of clinical skills lab training in preparing students for clinical practice.

For students to acquire practical skills, effective collaboration among all involved parties—students, universities, and training sites—is essential [38], along with sufficient opportunities to learn and practice new skills [37]. Since some students reported lower levels of perceived preparedness, supervising veterinarians would have had to compensate for this by taking up a teaching role [35]. For this purpose, the staff at ECR practices and clinics have to teach students while also maintaining the daily workflow and ensuring every patient receives adequate care. While it is generally clear to owners that animals presented to university clinics may be examined or treated by students for teaching purposes, this may not be equally evident in private practices, which could lead owners to decline student involvement. However, overall, students reported being rather satisfied with the teaching during their ECR. While instruction in therapy and pharmacology was rated lower than expected, a possible explanation could be that students were more involved in the diagnostic process of cases than in therapeutic planning. The latter is still somewhat surprising, given the central role of therapeutic decision-making and the use of pharmaceuticals in everyday clinical veterinary work. As with preparedness, students also rated the instruction of specific examinations and diagnostic imaging lower than expected. A possible explanation is that students may have missed opportunities to observe or take part in specific examinations if they spent their ECR at smaller practices focused on basic animal care. Such practices refer more complicated cases to other clinics for more advanced diagnostic imaging. However, this line of argument is contradicted by the fact that many students reported completing their placements in practices authorized for postgraduate training. In contrast, the lower rating of surgery is not very surprising. Especially if students do not feel very well prepared, it can be assumed that they would not be in a position to perform surgeries on their own. Furthermore, simpler surgeries—for example, castrating a male dog—might not happen every day, leaving fewer opportunities for students to practice them independently. It is understandable that more complex surgeries cannot be performed by students as they probably lack the understanding and skills necessary for those surgeries, and pose a higher risk of failure. On a positive note, surgical assistance was rated higher, as 60% of students felt well or very well instructed. Thus, students have the opportunity to see and assist in surgeries, thereby gaining a deeper under-standing and experience of surgical procedures, and hopefully will eventually be able to perform them independently.

### 4.4. Overall Satisfaction

In agreement with previous studies, students were mostly satisfied with the training and supervision during their ECR [7,13]. This is also in line with the results of the questions about the provision of knowledge and practical skills as these were rated mostly highly by students. The opportunity to work independently is something that matters to many students [17]. While over half of the students were satisfied with it during their ECR, it still ranked lowest in satisfaction, with 30% only moderately satisfied and 16% being not satisfied. As most supervising veterinarians had been in the same situation themselves after completing their veterinary studies and starting work in practice, they are able to assess the strengths and weaknesses of veterinary education [24], and can evaluate the skills of students and whether they are able to complete tasks independently. However, supervisors during ECR are primarily veterinarians, who might lack formal training in teaching methods. Often, instructors structure their teaching according to their own experience and use methods they found helpful during their own education [39]. As modern learners sometimes have different approaches to learning than previous generations [39], there is a potential conflict if students’ needs are not met. Since the students reported feeling rather poorly prepared, supervisors need to be careful not to overwhelm them, as stress and uncertainty lead to reduced engagement [35]. The implementation of a learning model could provide structure for both the instructor and the learner and, therefore, could result in a more beneficial outcome, as it provides guidance on how the training should be approached. Carr et al. [39] suggested the Five Microskills Model, which provides five steps to structure clinical education and can be used to enhance clinical thinking. As the model is rather flexible, it can be applied in different situations and to different learning types, making it suitable for students with varying levels of preparedness. One method used in human medicine to teach practical skills is the “see one, do one, teach one” method, in which the learner’s responsibility is gradually increased. Students first observe and receive explanations of a procedure, then perform it under supervision, and eventually guide others once they have gained sufficient experience [40]. It should be mentioned, though, that this method has raised concerns related to patient safety [40]. Therefore, competency must be ensured beforehand, and proper supervision is essential when students perform critical tasks.

## 5. Conclusions

A significant proportion of German veterinary students reported not feeling sufficiently prepared for the PY and their future career; however, they mostly rated the provision of theoretical knowledge and practical skills during ECR positively, highlighting the high significance of ECR for veterinary education. Although there is certainly room for improvement in some areas, as certain topics could be conveyed more effectively, the overall findings indicate that ECR can be regarded as an appreciated component of veterinary education. It should be mentioned, though, that this study is based on students’ subjective perceptions and does not directly reflect actual learning outcomes, and that the study cohort’s practical training before their PY may have been affected by COVID-19-related restrictions.

The study provides valuable insight into how ECR sites can attract students and optimize students’ ECR experience. For example, providing free accommodation or financial compensation can relieve students’ financial burden and thereby reduce stress. A direct supervisor, the use of a logbook, and established teaching methods can offer students structure and support, further reducing stress and promoting successful learning outcomes. Involving students in clinical workflows, teaching, letting them perform practical tasks, and encouraging independence are further aspects that are likely to be perceived positively and may further enhance students’ learning.

The findings of this study suggest that some students perceived room for improvement in the preparation they received prior to their ECR, particularly with regard to practical teaching and the integration of theoretical knowledge with clinical practice. In this context, the potential role of clinical skills labs in supporting students’ preparedness warrants further investigation.

Again, this study reflects only students’ perceptions and satisfaction, not objective learning outcomes; as such, no conclusions regarding the actual quality of teaching can be drawn. Therefore, further studies including objective methods to evaluate learning outcomes are needed. Further research is also needed on the actual quality of practical training at German universities, how it evolves, and whether future changes may influence students’ perceived preparedness for their PY. In addition, studies are needed on the establishment and implementation of EAEVE requirements, as well as how this subsequently affects the quality of teaching during ECR.

## Figures and Tables

**Table 1:** Students’ methods of finding their ECR placements (multiple responses possible; *n* = 386).

Method	%	*n*
Social media	20	76
Website of practices and clinics	61	237
Online job portals	24	93
Universities ^1^	22	85
Recommendations from other students	60	231
Recommendations from the bvvd e.V. ^2^	11	42
Listed bpt-accredited training practices ^3^	4	17
VMFT ^4^-Service-Center	0	0
Other	28	109

^1^ e.g., through newsletters or lectures by external practitioners. ^2^ bvvd e.V. = German Veterinary Students Association. ^3^ bpt = German Association of Veterinary Practitioners. ^4^ VMFT = German Veterinary Medicine Faculty Council.

## Data Availability

The original contributions presented in this study are included in the article and Supplementary Materials. Further inquiries can be directed to the corresponding author.

## Supplementary Materials

The following supporting information can be downloaded at: https://www.mdpi.com/article/10.3390/vetsci13070642/s1.

## Abbreviations

The following abbreviations are used in this manuscript:

CS	Chi-square test
EAEVE	European Association of Establishments for Veterinary Education
ECR	Extramural clinical rotation(s)
ESEVT SOP	European System of Evaluation of Veterinary Training Standard Operating Procedure
KW	Kruskal–Wallis test
MC	Multiple-choice-questions
PY	Practical Year
QA	Quality assurance
SC	Single-choice questions
VMFT	German Veterinary Medicine Faculty Council
VPH	Veterinary public health institutions
