# Supplementary material for: Student Perceptions of Preparation and Competency Development During Extramural Clinical Rotations in Germany: An Online Survey
Source: Vet Sci. 2026 Jun 30;13(7):642. doi: 10.3390/vetsci13070642 (PMC13431602; doi:10.3390/vetsci13070642)
Supplement: Supplementary file 1 [file vetsci-13-00642-s001.zip › Questionnaire_english.pdf]

## Demographics

What University do / did you study at?

- Berlin
- Giessen
- Hanover
- Leipzig
- Munich
- Outside of Germany

During what time period did you undertake your Practical Year?

- Before the winter semester 2022
- Winter semester 2022 / summer semester 2023 (final exams in 2024)
- Winter semester 2023 / summer semester 2024 (final exams in 2025)
- Winter semester 2024 / summer semester 2025 (final exams in 2026)
- My Practical Year is still to come

## Choosing ECR placements

In which animal species areas did you complete your ECR placements? Please ensure that the percentages total 100%. (Percentage allocation)

- Small animals
- Pet rodents
- Pet birds, reptiles and exotic pets
- Horses
- Ruminants
- Pigs
- Poultry
- Other

What criteria were important to you when choosing your ECR? (1 = very important; 5 = not important at all)

- Logbook
- ECR agreement
- Direct supervisor
- Positive experience from other students
- Free accommodation
- Proximity to home
- Privately organized accommodation (e.g. with familiy, friends etc.)
- Accessibility (e.g. by public transport, your own car)
- Compensation
- Non-monetary compensation (e.g. book presents, regular free lunch etc.)
- Own dog allowed at workplace
- Possibility to join in-house training (journal club, case discussions etc.)
- Access to literature
- Feedback meetings
- Authorization for continuing education
- Clinical focus that matches own interests
- Compliance with EAEVE regulations

If any other criteria were important to you when choosing ECR, you can list them here:

- Free text

Out of all your ECR placements during your Practical Year: What criteria were fulfilled during your ECR? (1 = was fulfilled at every ECR placement; 5 = was fulfilled at no ECR placement)

- Logbook
- ECR agreement
- Direct supervisor
- Free accommodation
- Compensation
- Non-monetary compensation (e.g. book presents, regular free lunch etc.)
- Own dog allowed at workplace
- Possibility to join in-house training (journal club, case discussions etc.)
- Access to literature
- Feedback meetings
- Authorization for continuing education
- Compliance with EAEVE regulations

If any other criteria were fulfilled during your ECR, you can list them here:

- Free text

Out of all your placements during the Practical Year: How did you look for your ECR or how did you become aware of the workplaces as possible ECR placements?

- Social media
- Websites of practices
- job.vet
- Other job portals (e.g. Vetstage)
- Universities (e.g. newsletters, lectures by external practitioners)
- Recommendations from other students
- Recommendations from the bvvd
- Listed bpt-accredited training practices
- VMFT-Service-Center
- Other: Free text

Which of the following applies? (1 = applies fully; 5 = does not apply at all)

- I was location bound when choosing my ECR placements
- I was dependent on compensation at my ECR placements
- When choosing my ECR placements I checked whether they were a potential future employer
- I used the Practical Year and the ECR as a period to reflect on what I career I want to choose after graduation
- Through my ECR my interest in future curative work was increased

## Experiences during ECR

To what extent were you able to complete all your ECR are preferred placements during your preferred time period?

- Fully
- Partially
- Not at all

To what extent did the Practical Year burden you financially? (1 = no burden at all; 5 = substantial burden)

- 1
- 2

- 3
- 4
- 5

How well prepared for the Practical Year did you feel concerning the following theoretical knowledge? (1 = very well; 5 = not at all)

- Propaedeutics (handling of animals, communication with owners, clinical examination)
- Knowledge of common diseases (symptoms, etiology, diagnostics)
- Therapy and Pharmacology (active substances, dosage, and dosage calculation)
- Surgery and anesthesiology (surgery methods, anesthesia induction and monitoring)

Out of all your ECR during your Practical Year: how well was this theoretical knowledge taught to you regardless of your prior knowledge? (1 = very well; 5 = not at all)

- Propaedeutics (handling of animals, communication with owners, clinical examination)
- Knowledge of common diseases (symptoms, etiology, diagnostics)
- Therapy and Pharmacology (active substances, dosage, and dosage calculation)
- Surgery and anesthesiology (surgery methods, anesthesia induction and monitoring)

How well prepared for the Practical Year did you feel concerning these practical skills? (1 = very well; 5 = not at all)

- General examination
- Specific examination (e.g. rectal examination of cattle, neurologic examination of dogs)
- Blood taking
- Application of drugs s.c., i.m.
- Diagnostic imaging
- Surgical assistance
- Performing small surgeries yourself (e.g. castration, disbudding)
- Professional handling of animals and communication with owners

Out of all your ECR during your Practical Year: how well were these practical skills taught to you regardless of your prior knowledge? (1 = very well; 5 = not at all)

- General examination
- Specific examination (e.g. rectal examination of cattle, neurologic examination of dogs)
- Blood taking
- Application of drugs s.c., i.m.
- Diagnostic imaging
- Surgical assistance
- Performing small surgeries yourself (e.g. castration, disbudding)
- Professional handling of animals and communication with owners

Concerning all your ECR during your Practical Year how satisfied were you concerning …? (1 = very satisfied; 5 = not satisfied at all)

- Team atmosphere
- Quality of teaching theoretical knowledge and practical skills
- The opportunity to work independently
- Supervision during ECR

Are there any other experiences during your Practical Year you would like to share)

- Free text
