# Supplementary material for: Student Perceptions of Preparation and Competency Development During Extramural Clinical Rotations in Germany: An Online Survey
Source: Vet Sci. 2026 Jun 30;13(7):642. doi: 10.3390/vetsci13070642 (PMC13431602; doi:10.3390/vetsci13070642)
Supplement: Supplementary file 1 [file vetsci-13-00642-s001.zip › Questionnaire_german.pdf]

Umfrage zu den extramuralen Praktika im Tiermedizin-Studium

Liebe (angehende) Kolleg:innen,
mein Name ist Sandra Kielmann. Derzeit schreibe ich meine Doktorarbeit am Institut für Veterinär-Epidemiologie und Biometrie an der Freien Universität Berlin. Darin möchten wir die Erwartungen und Wünsche zu den extramuralen kurativen Praktika im Tiermedizin-Studium von Seiten der Praktikumsgeber:innen und der (ehemaligen) Studierenden erfassen und vergleichen. Und genau dafür brauchen wir eure Hilfe!
Wenn ihr in den Jahren 2024 oder 2025 euren Abschluss gemacht habt oder nächstes Jahr fertig werdet, würden wir uns sehr freuen, wenn ihr euch kurz die Zeit nehmt, an der Umfrage teilzunehmen.
Die Bearbeitungsdauer beträgt etwa 5-10 Minuten.
Bitte gebt in den Freitextfeldern keine persönlichen Informationen ein, die einen Rückschluss auf eure Person oder eure Praktikumsstätte zulassen. Alle Daten werden anonym erhoben und können eurer Person nicht zugeordnet werden.
Es besteht die Möglichkeit, am Ende dieser Umfrage an einem Gewinnspiel teilzunehmen. Zu Verlosen sind ein Thermobecher vom TVD UniTeam und zwei Wunschgutscheine.
Die Ergebnisse dieser Umfrage werden außerdem im Rahmen eines Praktikumsreportes vom TVD – Partner für Tierärzte veröffentlicht.

Vielen Dank für eure Unterstützung unserer Studie!
Sandra Kielmann (Doktorandin)
Prof. Dr. Roswitha Merle
Dr. Charlotte Jensen

1. An welcher Universität studierst du / hast du studiert? (SC)
    a. Berlin
    b. Gießen
    c. Hannover
    d. Leipzig
    e. München
    f. Außerhalb von Deutschland
2. In welcher Zeit warst du im Praktischen Jahr? (SC)
    a. Vor WS 2022
    b. WS 2022 / SoSe 2023 (Staatsexamen 2024)
    c. WS 2023 / SoSe 2024 (Staatsexamen 2025)
    d. WS 2024 / SoSe 2025 (Staatsexamen 2026)
    e. Das Praktische Jahr steht mir noch bevor
3. Bei welchen Tierarten hast du deine kurativen Praktika absolviert? Bitte darauf achten, dass es zusammengerechnet 100 % ergibt! (Prozentfrage, 5er-Schritte)
    a. Kleintiere
    b. Heimtiere
    c. Ziervögel, Reptilien, Exoten
    d. Pferde
    e. Wiederkäuer
    f. Schweine
    g. Geflügel
    h. Andere

4. Welche Kriterien waren dir bei der Auswahl deiner kurativen Praktika wichtig? (Likert Skala)

	1 – sehr wichtig	2	3	4	5 – gar nicht wichtig	Keine Angabe
Ein strukturiertes Praktikumsprogramm / Praktikumsleitfaden						
Eine schriftliche Praktikumsvereinbarung						
Ein:e direkte:r Ansprechpartner:in / Betreuer:in für das Praktikum						
Positive Erfahrungsberichte von bisherigen Praktikant:innen						
Eine kostenlose Unterkunft						
Nähe zum Heimatort						
Möglichkeit einer privat organisierten kostenlosen Unterkunft (z.B. bei Familie, Freund:innen etc.)						
Erreichbarkeit (z.B. mit öffentlichen Verkehrsmitteln, dem eigenen Auto)						
Eine Vergütung						
Nicht-monetäre Vergütung (z.B. in Form von Fachbüchergeschenken, regelmäßiges kostenloses Mittagessen etc.)						
Der eigene Hund darf mit zur Arbeit gebracht werden						
Möglichkeit zur Teilnahme an praxisinternen Weiterbildungen (Journal Club, Fallbesprechungen etc.)						
Zugang zu Fachliteratur						
Feedback-Gespräche						
Die Arbeitsstätte hat eine:n Fachtierärzt:in mit Weiterbildungsermächtigung						
Bestimmte fachliche Schwerpunkte, die meinen eigenen Interessen entsprechen						
Die Arbeitsstätte erfüllt die Anforderungen der EAEVE						

Infos zu den Anforderungen der EAEVE: Auf dem Veterinärmedizinischen Fakultätentag im Juli 2024 wurde entschieden, dass zukünftig alle Einrichtungen, in denen Praktika nach Vorgabe der TAppV absolviert werden, einen bestimmten Qualitätsstandard einhalten sollen. Hierzu müssen die betreuenden Tierärzt:innen an einer didaktischen Qualifizierungsmaßnahme teilgenommen haben.
Weitere Infos: https://www.vmft.de/_medien/Informationsschreiben_Vet-Service-250724.pdf

5. Falls dir noch weitere Kriterien bei der Auswahl deiner kurativen Praktika wichtig waren, die bisher nicht aufgelistet wurden, kannst du sie hier angeben:
    a. Freitext (freiwillig)

6. Von all deinen Praktika im Praktischen Jahr: Welche dieser Kriterien wurden bei deinen kurativen Praktika erfüllt? (Likert Skala)

	1 – wurde bei jedem Praktikum erfüllt	2	3	4	5 – wurde bei keinem Praktikum erfüllt	Keine Angabe
Ein strukturiertes Praktikumsprogramm / Praktikumsleitfaden						
Eine schriftliche Praktikumsvereinbarung						
Ein:e direkte:r Ansprechpartner:in / Betreuer:in für das Praktikum						
Eine kostenlose Unterkunft						
Eine Vergütung						
Nicht-monetäre Vergütung (z.B. in Form von Fachbüchergeschenken, regelmäßiges kostenloses Mittagessen etc.)						
Der eigene Hund darf mit zur Arbeit gebracht werden						
Möglichkeit zur Teilnahme an praxisinternen Weiterbildungen (Journal Club, Fallbesprechungen etc.)						
Zugang zu Fachliteratur						
Feedback-Gespräche						
Die Arbeitsstätte hat eine:n Fachtierärzt:in mit Weiterbildungsermächtigung						
Die Arbeitsstätte erfüllt die Anforderungen der EAEVE						

Infos zu den Anforderungen der EAEVE: Auf dem Veterinärmedizinischen Fakultätentag im Juli 2024 wurde entschieden, dass zukünftig alle Einrichtungen, in denen Praktika nach Vorgabe der TAppV absolviert werden, einen bestimmten Qualitätsstandard einhalten sollen. Hierzu müssen die betreuenden Tierärzt:innen an einer didaktischen Qualifizierungsmaßnahme teilgenommen haben.
Weitere Infos: https://www.vmft.de/_medien/Informationsschreiben_Vet-Service-250724.pdf

7. Falls noch weitere Kriterien bei deinen kurativen Praktika erfüllt wurden, die bisher nicht aufgelistet waren, kannst du sie hier eingeben:
    a. Freitext (freiwillig)
8. Bezogen auf all deine Praktika im Praktischen Jahr: Wie hast du nach deinen kurativen Praktikumsplätzen gesucht bzw. wie bist du auf die Arbeitsstätten als mögliche Praktikumsgeber aufmerksam geworden? (MC)
    a. Social media

b. Praxiswebseiten
c. job.vet
d. Andere Jobwebseiten (z.B. Vetstage)
e. Universitäten (z.B. Newsletter, Vorlesungen von externen etc.)
f. Empfehlungen anderer Studierender
g. Praktikumsempfehlungen des bvvd
h. Liste der bpt-Ausbildungspraxen
i. Liste des VMFT-Service-Center
j. Sonstiges: Freitext

9. Was trifft zu? (Likert Skala)

	1 – trifft voll zu	2	3	4	5 – trifft gar nicht zu
Ich war bei der Auswahl meiner kurativen Praktika ortsgebunden					
Ich war auf eine Vergütung bei meinen kurativen Praktika angewiesen					
Ich habe bei der Auswahl meiner kurativen Praktika bereits darauf geachtet, ob sie als spätere Arbeitgebende in Frage kommen					
Ich habe das Praktische Jahr und die kurativen Praktika in der Zeit noch genutzt, um mir klar zu werden, was ich nach meinem Universitätsabschluss beruflich machen möchte					
Durch meine Praktika in kurativen Arbeitsstätten wurde mein Interesse an einer späteren Tätigkeit in der kurativen Tiermedizin gesteigert					

Erfahrungen in den Praktika

Einige Studierenden machen sich schon länger Gedanken, in welchen Arbeitsstätten sie gerne ihre kurativen Praktika absolvieren wollen und haben dabei teilweise auch Wunschzeiträume oder müssen mehrere Wunschpraktika zeitlich miteinander abstimmen. Da in manchen Arbeitsstätten die Praktikumsplätze limitiert sind, ist es aber nicht immer möglich, alle Wunschpraktika zu den gewünschten Zeiträumen zu absolvieren oder überhaupt bei allen einen Praktikumsplatz zu bekommen.

10. Inwiefern konntest du all deine Wunschpraktika zu deinen Wunschzeiträumen absolvieren? (SC)
    a. Voll und ganz
    b. Teilweise
    c. Gar nicht
11. Inwieweit hat das Praktische Jahr dich finanziell belastet? (1 = keine Belastung; 5 = sehr starke Belastung) (SC)

a. 1
b. 2
c. 3
d. 4
e. 5

12. Wie fühltest du dich vor Beginn des Praktischen Jahres in folgenden **theoretischen Kenntnissen vorbereitet?** (Likert Skala)

	1 – gut vorbereitet	2	3	4	5 – gar nicht vorbereitet
Propädeutik (Umgang mit Patiententieren, Kommunikation mit Besitzer:innen, klinische Untersuchung)					
Kenntnisse bzgl. geläufiger Krankheiten (Symptome, Ätiologie, Diagnostik)					
Therapie bzw. Pharmakologie (Wirkstoffe, Berechnung, Dosierung)					
Chirurgie und Anästhesie (OP-Methoden, Narkoseeinleitung/-überwachung)					

13. Von all deinen Praktika im Praktischen Jahr: Inwiefern wurden dir die **theoretischen Kenntnisse** unabhängig von dem eigenen Kenntnisstand in deinen kurativen Praktika **vermittelt?** (Likert Skala)

	1 – sehr viel	2	3	4	5 – gar nicht
Propädeutik (Umgang mit Patiententieren, Kommunikation mit Besitzer:innen, klinische Untersuchung)					
Kenntnisse bzgl. geläufiger Krankheiten (Symptome, Ätiologie, Diagnostik)					
Therapie bzw. Pharmakologie (Wirkstoffe, Berechnung, Dosierung)					
Chirurgie und Anästhesie (OP-Methoden, Narkoseeinleitung/-überwachung)					

14. Wie fühltest du dich vor Beginn des Praktischen Jahres in folgenden **praktischen Fähigkeiten vorbereitet?** (Likert Skala)

	1 – sehr gut vorbereitet	2	3	4	5 – gar nicht vorbereitet
Durchführung einer allgemeinen Untersuchung					

Durchführung einer speziellen Untersuchung (z.B. Rind rektalisieren, Hund neurologisch untersuchen)					
Blutentnahme					
Medikamentenapplikation s.c., i.m.					
Durchführung bildgebender Verfahren					
Assistenz bei Operationen					
Selbstständiges Durchführen kleiner Eingriffe (z.B. Kastration, Enthornung)					
Freundlicher und professioneller Umgang mit Patienten und Besitzer:innen					

15. Von all deinen Praktika im Praktischen Jahr: Inwiefern wurden dir die **praktischen Fähigkeiten** unabhängig von dem eigenen Kenntnisstand in deinen kurativen Praktika **vermittelt?** (Likert Skala)

	1 – sehr viel	2	3	4	5 – gar nicht
Durchführung einer allgemeinen Untersuchung					
Durchführung einer speziellen Untersuchung (z.B. Rind rektalisieren, Hund neurologisch untersuchen)					
Blutentnahme					
Medikamentenapplikation s.c., i.m.					
Durchführung bildgebender Verfahren					
Assistenz bei Operationen					
Selbstständiges Durchführen kleiner Eingriffe (z.B. Kastration, Enthornung)					
Freundlicher und professioneller Umgang mit Patienten und Besitzer:innen					

16. Wie zufrieden warst du zusammenfassend in deinen kurativen Praktika im Praktischen Jahr im Hinblick auf...? (Likert Skala)

	1 – sehr zufrieden	2	3	4	5 – gar nicht zufrieden
Die Arbeitsatmosphäre im Team					
Die Vermittlung von tierärztlichem Wissen und Fähigkeiten					

Die Möglichkeit, selbstständig Aufgaben auszuführen					
Die Betreuung während der Praktika					

17. Möchtest du uns noch weiteres zu deinen Erfahrungen im Praktischen Jahr mitteilen?
    a. Freitext (freiwillig)

SC = Single Choice Frage
MC = Multiple Choice Frage
