# Supplementary material for: Student Perceptions of Preparation and Competency Development During Extramural Clinical Rotations in Germany: An Online Survey
Source: Vet Sci. 2026 Jun 30;13(7):642. doi: 10.3390/vetsci13070642 (PMC13431602; doi:10.3390/vetsci13070642)
Supplement: Supplementary file 1 [file vetsci-13-00642-s001.zip › s3_supplementary-tables-and-figures.pdf]

Supplementary table S1. Cohort comparison of perceived financial burden during the Practical Year

	PY 2022/23	PY 2023/24	PY 2024/25	p
Financial burden	3 (2-4)	4 (3-4.5)	4 (3-5)	0.046

Data are presented as median (25th–75th percentile). Lower values indicate lower financial burden (1 = no burden, 5 = substantial burden). Differences between cohorts were assessed using the Kruskal–Wallis test.

Supplementary table S2. Cohort comparison of perceived theoretical and practical preparedness and teaching

	PY 2022/23	PY 2023/24	PY 2024/25	p
**Prepared**				
Propaedeutics	3 (2-3.5)	3 (2-3)	2 (2-3)	0.031
Diseases	3 (2-4)	3 (2-3,5)	3 (2-3)	0.014
Therapy & Pharmacology	4 (3-4)	4 (3-4)	3 (3-4)	< 0.001
Surgery & Anaesthesia	4 (3-4)	4 (3-4)	4 (3-4)	0.060
**Taught**				
Propaedeutics	2 (1-3)	2 (1-3)	2 (1-2)	0.045
Diseases	2 (2-3)	2 (1-3)	2 (1-2)	0.022
Therapy & Pharmacology	3 (2-4)	3 (2-4)	2 (2-3)	0.005
Surgery & Anaesthesia	3 (2-3)	2 (2-3)	2 (2-3)	0.055
**Prepared**				
General examination	2 (1-3)	2 (2-3)	2 (1-3)	0.012
Specific examination	4 (3-5)	4 (3-5)	3 (2.5-4)	0.007
Blood taking	3 (2.5-4)	3 (2-4)	3 (2-4)	0.004
Application of drugs	2 (1.5-3)	2 (1-3.5)	2 (1-3)	0.061
Diagnostic imaging	4 (3-5)	4 (3-5)	3 (2-4)	< 0.001
Surgical assistance	4 (3-5)	4 (3-5)	4 (2-5)	0.035
Surgery	5 (4-5)	5 (4-5)	4 (3-5)	< 0.001
Handling & communication	3 (2-4)	3 (2-4)	2 (2-4)	0.003
**Taught**				
General examination	2 (1-2.5)	2 (1-2)	1 (1-2)	0.109
Specific examination	2 (2-3.5)	2 (2-3)	2 (1-3)	0.077
Blood taking	2 (1-3)	2 (1-3)	1 (1-2)	< 0.001
Application of drugs	1 (1-2)	1 (1-2)	1 (1-2)	0.288
Diagnostic imaging	3 (2-3)	3 (2-3)	2 (1-3)	< 0.001
Surgical assistance	2 (2-3)	2 (2-3)	2 (1-3)	0.100
Surgery	3 (2-4)	3 (2-4)	3 (2-4)	0.202
Handling & communication	2 (1-3)	2 (1-3)	2 (1-3)	0.004

Data are presented as median (25th–75th percentile). Lower values indicate higher perceived preparedness/teaching quality (1 = very well prepared/very good teaching, 5 = not at all prepared/very poor teaching). Differences between cohorts were assessed using the Kruskal–Wallis test.

Supplementary table S3. Cohort comparison of students' satisfaction with supervision and working environment during their ECR

	PY 2022/23	PY 2023/24	PY 2024/25	p
Team atmosphere	2 (1-3)	2 (1-3)	2 (1-2)	0.420

Quality of teaching during ECR	2 (2-3)	2 (1-3)	2 (1-2)	0.038
Opportunity to work independently	3 (2-3.5)	3 (2-3)	2 (2-3)	0.143
Supervision during ECR	2 (2-3)	2 (2-3)	2 (1-3)	0.400

Data are presented as median (25th–75th percentile). Lower values indicate higher satisfaction (1 = very satisfied, 5 = not satisfied at all). Differences between cohorts were assessed using the Kruskal–Wallis test.

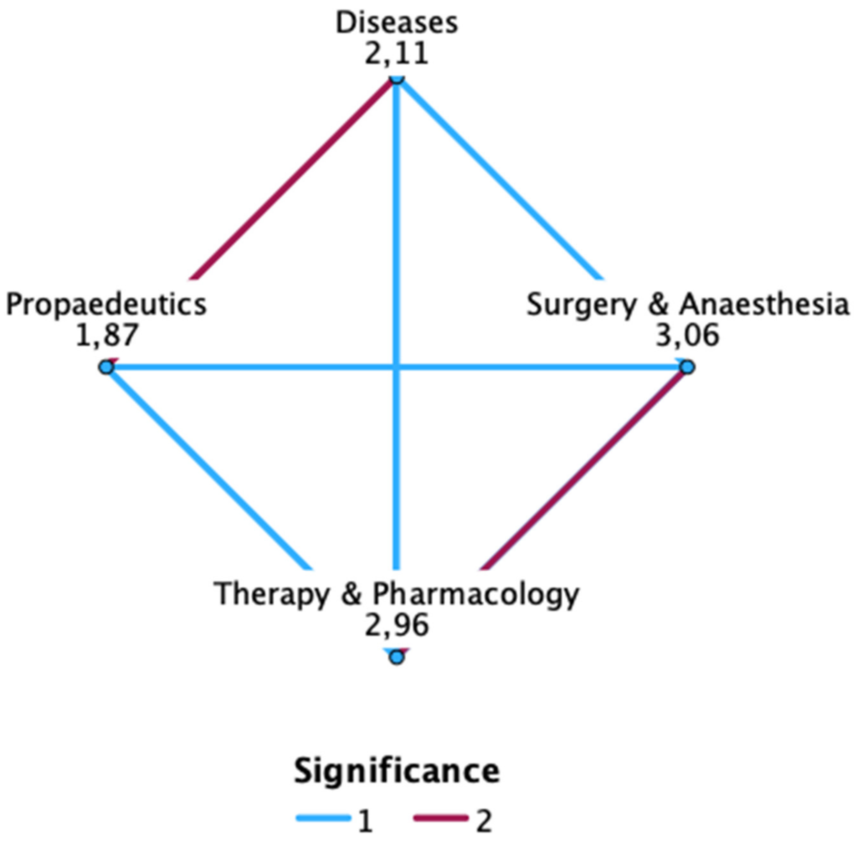

Supplementary figure S1. Post-hoc comparisons following Friedman's rank test for perceived preparedness in terms of theoretical knowledge. Blue (1) indicates a significant difference and red (2) indicates a non-significant difference after Bonferroni adjustment.

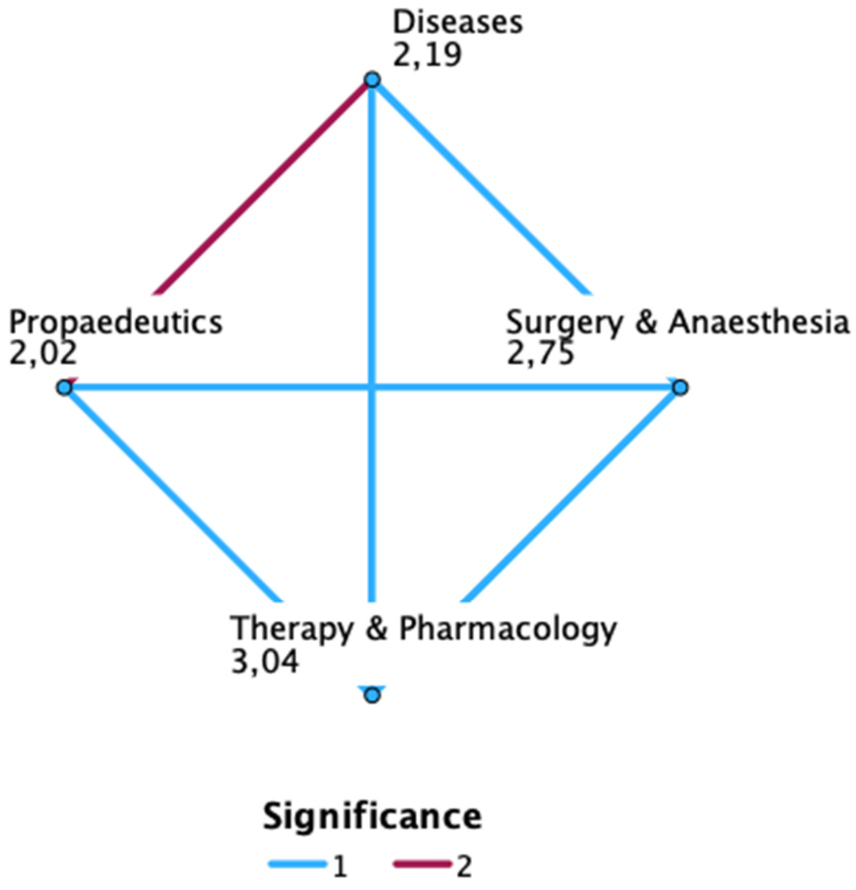

Supplementary figure S2. Post-hoc comparisons following Friedman's rank test for perceived teaching of theoretical knowledge during ECR. Blue (1) indicates a significant difference and red (2) indicates a non-significant difference after Bonferroni adjustment.

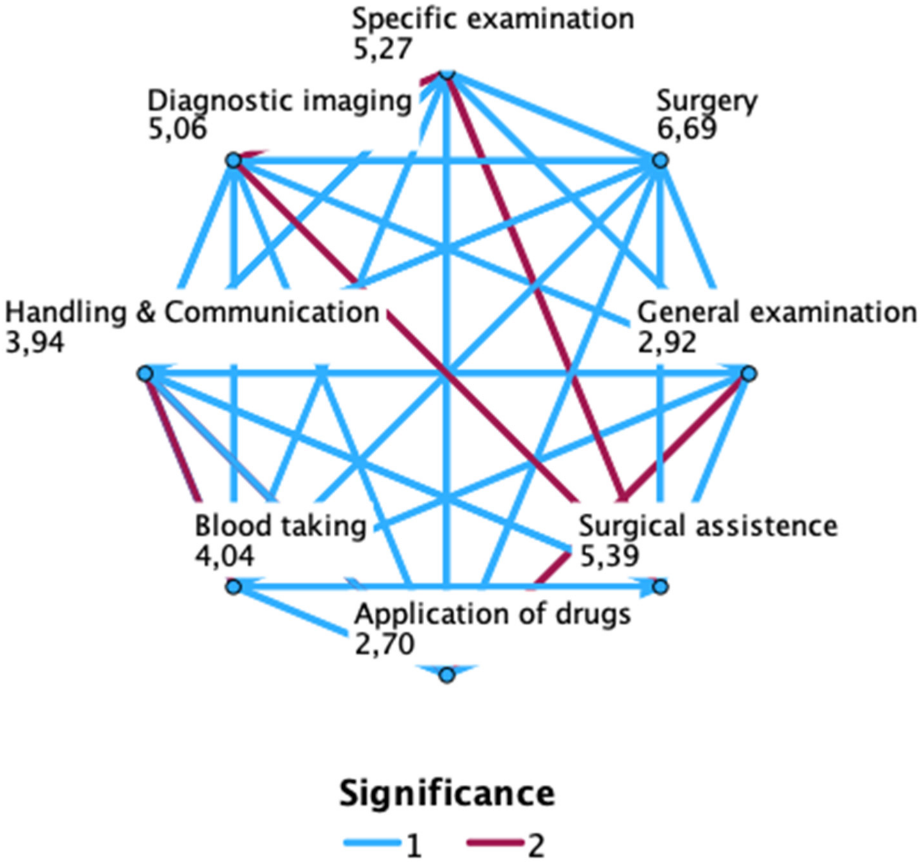

Supplementary figure S3. Post-hoc comparisons following Friedman's rank test for perceived preparedness in terms of practical skills. Blue (1) indicates a significant difference and red (2) indicates a non-significant difference after Bonferroni adjustment.

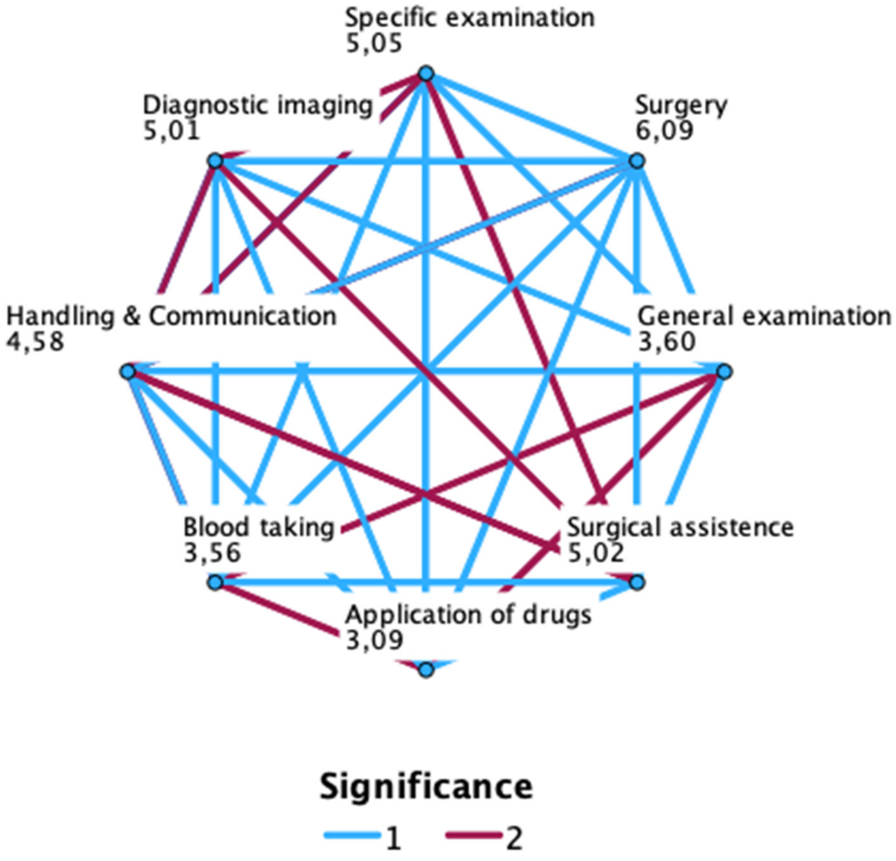

Supplementary figure S4. Post-hoc comparisons following Friedman's rank test for perceived teaching of practical skills during ECR. Blue (1) indicates a significant difference and red (2) indicates a non-significant difference after Bonferroni adjustment.
